# Evaluation of the Pathways for Survivors Program to Address Breast Cancer Survivorship–Associated Distress: Survey Study

**DOI:** 10.2196/31756

**Published:** 2022-02-25

**Authors:** Saumya Umashankar, Matina Elise Mamounas, Madeline Matthys, Edward Kenji Hadeler, Emily Claire Wong, Greg Hicks, Jimmy Hwang, Amy Jo Chien, Hope S Rugo, Deborah Hamolsky, Laura Esserman, Michelle Melisko

**Affiliations:** 1 Department of Medicine University of California San Francisco San Francisco, CA United States; 2 Foster, Hicks & Associates San Francisco, CA United States

**Keywords:** breast cancer, depression, anxiety, quality of life, breast cancer survivors, cancer survivorship, mental health, psychological health

## Abstract

**Background:**

Patients with breast cancer frequently experience escalation of anxiety after completing curative treatment.

**Objective:**

This study evaluated the acceptability and psychological impact of a 1-day workshop to emphasize behavioral strategies involving intention and self-efficacy.

**Methods:**

Breast cancer survivors who attended a 1-day Pathways for Survivors workshop provided feedback and completed electronic quality of life (QOL) questionnaires at baseline, 1 and 6 weeks, and 6 months after the workshop. Attendees’ baseline QOL scores were compared to follow-up (FUP) scores. Scores from patients receiving routine FUP care were also compiled as a reference population.

**Results:**

In total, 77 patients attended 1 of 9 workshops. The mean satisfaction score was 9.7 out of 10 for the workshop and 9.96 out of 10 for the moderator. Participants’ baseline mean Patient-Reported Outcomes Measurement Information System (PROMIS) anxiety and depression scores were 57.8 (SD 6.9) and 55.3 (SD 7.5), respectively, which were significantly higher than those of patients receiving routine FUP care (49.1, SD 8.3 and 47.3 SD 8.0, respectively). PROMIS anxiety and depression scores decreased, and the Happiness Index Profile (HIP-10) score—measuring intention and resiliency—increased significantly at 1- and 6-week FUPs.

**Conclusions:**

The Pathways for Survivors program was favorably received. Anxiety and depression decreased significantly at 1- and 6-weeks after the workshop and remained below baseline at 6 months. Increased HIP-10 scores suggest that patients acquired and implemented skills from the workshop. A 1-day workshop led by a lay moderator significantly improved several psychological measures, suggesting that it may be a useful and time-efficient strategy to improve QOL in breast cancer survivors. We are investigating whether an abbreviated “booster” of the intervention at a later date could further improve and maintain QOL gains.

## Introduction

There were an estimated 3.8 million breast cancer survivors in the United States in 2019, and this number is expected to be close to 5 million by 2030 [1]. Transitioning from a patient with cancer to a cancer survivor is challenging, and many patients with breast cancer have unmet physical and emotional needs [2-5]. Studies have found increased rates of anxiety and depression among breast cancer survivors over the short and long term, and these problems appear more prominent in younger survivors and those with pre-existing psychological symptoms [4,6,7]. Many studies also identify fear of cancer recurrence (FCR) and difficulty in returning to “normalcy” as potential sources of distress in this population [4,8,9]. The nature of intrusive thoughts associated FCR have been shown to share many characteristics with worry or anxiety [10]. Moreover, a systematic review of adult cancer survivors found that depression and anxiety were significantly correlated with FCR, and psychological distress is a strong predictor of FCR [11]. During the acute phase of care when attending regular medical appointments, patients often feel more secure that there is active monitoring for signs and symptoms of cancer recurrence. After active treatment ends, patients with breast cancer may feel a loss of a safety net. A comprehensive review of breast cancer survivors (≥1 year from diagnosis) showed compelling evidence of an increased risk of anxiety, depression, suicide, and neurocognitive and sexual dysfunction in breast cancer survivors compared with women with no prior cancer [6]. These findings indicate the need for novel interventions to help manage these psychological symptoms in breast cancer survivors.

The Pathways for Survivors program was developed through a collaboration between the moderator (GH) and clinicians (MM, HR, DH, and LE) at the University of California San Francisco (UCSF). The basic principles and content of the Pathways workshop are based on a positive psychology model of cognitive behavioral therapy (CBT). This model, consistent with the Broaden-and-Build theory of positive psychology, suggests that experiencing positive emotions broadens a person’s awareness and encourages varied and novel thoughts and actions, which, in turn, strengthens the individual’s personal skills and resources [12]. Multiple CBT interventions have been shown to decrease anxiety and depression in various breast cancer populations [13-17].

The Pathways for Survivors program teaches specific techniques for increasing positive emotions on a daily basis, equipping patients with a variety of skills and tools to improve their quality of life (QOL) in the context of life-limiting illness. The intervention is based on a system of 9 behaviors that have been shown in other contexts to enhance QOL and emotional well-being [18,19]. With the aid of grant and philanthropic funding, the UCSF Breast Care Center (BCC) has offered Pathways workshops several times a year since 2015 as a free resource to breast cancer survivors, with a focus on patients who recently completed active treatment. Qualitative and quantitative feedback on the acceptability and utility of the Pathways for Survivors program has been collected for quality improvement purposes, allowing us to better characterize the acceptability and psychological impact of the workshop. We hypothesized that this day-long workshop would have favorable effects on patient QOL by reducing both short- and long-term anxiety and depression.

This study aimed to assess the impact of the Pathways for Survivors program, a 1-day layperson-led workshop for breast cancer survivors, on breast cancer survivors’ psychological distress as evaluated by a number of standardized measures of anxiety and depression. We characterized how patients received the intervention and evaluated the change in measures of patients’ psychological distress from before to after the intervention.

## Methods

### Ethics Approval

Approval was obtained from the UCSF ethics committee (15-17099). The procedures used in this study adhere to the tenets of the Declaration of Helsinki. Informed consent was obtained from all individual participants included in the study. Patients provided written informed consent for publishing their deidentified data.

### Methods Overview

Patients with stage 0-3 breast cancer, who had at least one clinic visit at the UCSF BCC and had completed their acute phase of care, including chemotherapy and breast surgery, were considered eligible and were invited to attend a day-long Pathways for Survivors workshop. Patients were recruited by their medical oncologist or breast surgeon through flyers posted in UCSF clinics, and at local breast cancer survivorship and supportive care events. For the last 2 sessions, workshops were limited to patients aged 50 years and under since the philanthropic funding to support these two workshops was intended to focus on “younger” breast cancer survivors. The workshops were conducted on a weekend day, lasting from approximately 8:30 AM to 4 PM with a 45-minute lunch break during which patients were encouraged to engage in informal interaction. The workshops were moderated by author GH and included a series of 9 lessons or exercises, most of which required substantial interaction among the participants. The central framework of the workshop was centered on “intention,” which is defined in this program as “making a conscious choice toward the most beneficial thought, feeling, or behavior.” Other exercises were based on the concepts of truth, accountability, identification, centrality, recasting, options, appreciation, and giving. Upon completion of the workshop, participants were asked to complete anonymous feedback surveys on program content and moderator quality.

Attendees were asked to complete a series of electronic surveys via the REDCap system at baseline (before the day-long program), 1 week after the workshop, and 6 weeks after the workshop. For the last 4 workshops, a 6-month follow-up (FUP) survey was added. Within the questionnaire, patients were presented with a consent section to have their data used for research purposes. However, patients could opt to participate in the workshop and opt out of data-sharing. Specific survey measures included the National Cancer Institute’s PROMIS (Patient-Reported Outcomes Measurement Information System) anxiety and depression short-form questionnaire and the Happiness Index Profile (HIP-10) scale—a measure of psychological intention and resiliency.

The PROMIS anxiety and depression scales are two independent short-form, 4-item questionnaires that assess self-reported anxiety and depression in the past 7 days. Each item is scored from 1 (never) to 5 (always), with higher scores indicating greater anxiety or depression. PROMIS instruments were graded with item-level calibrations using the Health Measures Scoring Service [20] to determine PROMIS anxiety and depression T-scores.

The HIP-10 (previously HI/P6 scale) is a 10-item questionnaire assessing positive affect, intention, and resiliency. Each item is scored from 0 (strongly disagree) to 10 (strongly agree). HIP-10 scores are calculated by adding the scores for each item to generate a total score out of 100, and an increase in the score suggests greater uptake of the “intention” model. Through an independent, unpublished, pilot validation analysis in a population including college students, employees of large corporations, and retirees, the HIP-10 was found to have high internal consistency (Cronbach α=.847) and correlation with the POMS (Profiles of Moods) total scale and multiple subscales.

Within the UCSF BCC, all new patients are asked to complete an intake survey that includes demographic information, health history, and QOL instruments including PROMIS anxiety and depression. We have also implemented electronic delivery of follow-up surveys to early-stage patients in ongoing routine care. A subset of these patients agreed to have their survey data used for research. To better contextualize the Pathways patients’ baseline scores within a broader general population of early-stage FUP patients at the UCSF BCC, we utilized data from patients who had completed an FUP survey, did not attend Pathways, and consented to have their data used for research. Hereinafter, these patients are referred to as the “comparison group.”

The primary goal of this study was to evaluate longitudinal change in patient-reported psychological distress measures, including PROMIS depression and anxiety and HIP-10, and to evaluate demographic and clinical covariates within this population, which may help predict patients who would benefit most from this intervention.

We also compared baseline PROMIS anxiety and depression scores as well as demographic and clinical descriptors of the Pathways patients to a comparison group of early-stage FUP patients along with their PROMIS anxiety and depression scores collected at a single FUP survey.

### Statistical Analysis

Descriptive statistics were used to summarize demographic and clinical data including age, stage, hormone receptor and HER2 status, nodal status, and time from diagnosis to completion of the baseline survey for Pathways participants. An independent samples *t* test and chi-square tests were conducted to compare demographics for Pathways participants and the comparison group. Independent 2-sample *t* tests were also used to compare the one-time scores on the PROMIS anxiety and depression scales of the comparison group to baseline scores of Pathways participants.

For Pathways participants, paired samples *t* tests were used to compare the PROMIS anxiety, PROMIS depression, and HIP-10 scores between baseline and the 1-week, 6-week, and 6-month scores for significance. Two-tailed *P* values of <.05 were considered significant. Among Pathways participants, analyses were conducted using paired samples *t* tests to determine if factors including age, stage, nodal status, hormone receptor status, and time from diagnosis were associated with the change from baseline in PROMIS and HIP-10 scores at 1 week, 6 weeks, and 6 months. All *t* tests used in this study are 2-tailed.

### Availability of Data and Material

The data sets generated and analyzed during this study are available from the corresponding author on reasonable request.

## Results

Nine sessions were held between September 2015 and December 2019. In total, 79 patients participated in the Pathways workshop and provided feedback on their satisfaction with the day-long session. A total of 77 patients consented to have their QOL data (including PROMIS and HIP-10 scores) used for research. Overall, 71 patients completed at least 1 FUP survey, of whom, 68 completed the 1-week FUP (completion rate=88%) and 61 completed the 6-week FUP (completion rate=80%). The 6-month FUP survey was sent to participants from the last 4 workshops. Of the 50 patients invited to complete the 6-month survey, 32 completed it (completion rate=65%).

Demographic data for Pathways participants and the routine follow-up comparison group patients who agreed to use of their clinically collected data for research are presented in Table 1. Pathways participants were younger than the routine FUP care patients (mean age 51.3 vs 58.5 years, *P*<.001). There were no significant differences in stage, hormone receptor and HER2 status, or nodal status. Pathways participants were, on average, 1.5 years from their diagnosis. The majority of participants were White (75.3%), well-educated (college graduates or above, 92%), and employed (45% full-time and 22% part-time).

Pathways participants had significantly higher baseline PROMIS anxiety and depression scores than the scores from a single FUP time point in the routine FUP comparison group. The baseline PROMIS anxiety mean score was 57.8 (SD 6.9) for Pathways patients versus 49.1 (SD 8.3) for the comparison group patients. Similarly, the baseline PROMIS depression mean score was 55.3 (SD 7.5) for Pathways patients versus 47.3 (SD 8) for the comparison group patients (*P*<.001 for both comparisons).

**Table 1:** Demographic and clinical characteristics of Pathways participants (N=77) and early-stage routine follow-up care patients (N=71).

Characteristics		Pathways participants	Routine follow-up care patients	*P* value			
Age (years), mean (SD; median)		51.4 (10.74; 51.3)	58.5 (11.79; 59.0)	<.001			
**Stage, n (%)**					.30		
	0 or 1	31 (40.3)	23 (32.4)				
	2 or 3	46 (59.7)	48 (67.6)				
**Hormone receptor status, n (%)**					.13		
	Negative	13 (16.9)	6 (8.5)				
	Positive	64 (83.1)	65 (91.5)				
**HER2 status, n (%)**					.30		
	Negative	61 (79.2)	51 (71.8)				
	Positive	16 (20.8)	20 (28.2)				
**Nodal involvement, n (%)**					.06		
	No	50 (64.9)	36 (50.7)				
	Yes	26 (33.8)	35 (49.3)				
**Treatment length, n (%)**					N/A^a^		
	<6 months	22 (28.6)	—^b^				
	≥6 months	55 (71.4)	—				
**Race, n (%)**					N/A		
	White	58 (75.3)	—				
	Asian	11 (14.3)	—				
	Other	6 (8)	—				
	Not reported	2 (2.6)	—				
**Education, n (%)**					N/A		
	Some high school or less	0 (0)	—				
	High school graduate or graduate equivalency degree	1 (1.3)	—				
	Some college or technical school	5 (6.5)	—				
	College graduate	26 (33.8)	—				
	Some graduate school	2 (2.6)	—				
	Master’s degree	30 (39.0)	—				
	PhD, MD, JD, or other	13 (16.9)	—				
**Employment status**					N/A		
	Full-time (≥35 hours/week)	35 (45.5)	—				
	Part-time (<35 hours/week)	17 (22.1)	—				
	Full-time parenting or caregiving	4 (5.2)	—				
	Student	1 (1.3)	—				
	Retired	8 (10.4)	—				
	On leave/disability	7 (9.1)	—				
	Other	5 (6.5)	—				
**Annual income (US $), n (%)**					N/A		
	<25,000	8 (10.5)	—				
	25,000-49,999	4 (5.3)	—				
	50,000-74,999	10 (13.2)	—				
	75,000-99,999	11 (14.5)	—				
	>100,000	43 (56.6)	—				
**Health insurance, n (%)**					N/A		
	Health insurance through employer	42 (54.5)	—				
	Health insurance through partner’s employer	18 (23.4)	—				
	Private health insurance	11 (14.3)	—				
	Medi-Cal/Medicare/Medicaid or some other public coverage	6 (7.8)	—				
**Marital status**					N/A		
	Married	46 (59.7)	—				
	In a committed relationship	11 (14.3)	—				
	Single	12 (15.6)	—				
	Divorced/separated	8 (10.4)	—				

^a^N/A: not applicable.

^b^—: not available.

**Figure 1:**
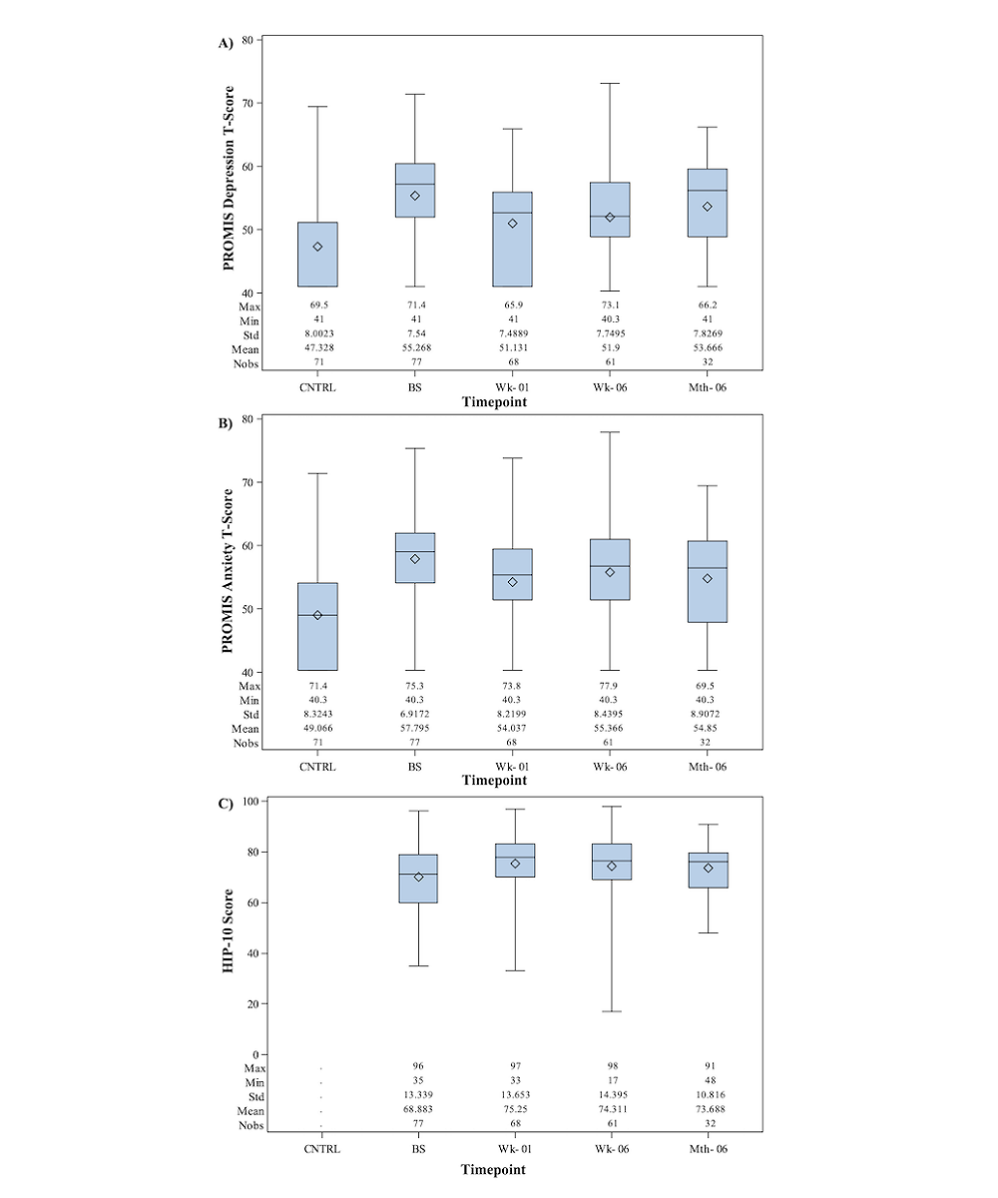
Distribution of (A) PROMIS depression T-scores, (B) PROMIS anxiety T-scores, and (C) HIP-10 scores at baseline (participants and comparison group) and follow up (participants only). BS: baseline; CNTRL: baseline comparison group; HIP-10: Happiness Index Profile; Nobs: number of observations; PROMIS: Patient-Reported Outcomes Measurement Information System.

The distribution of PROMIS depression and anxiety and HIP-10 scores over time are depicted in Figure 1. PROMIS anxiety scores decreased significantly at 1 week (mean difference 3.884, SD 6.616; *P*<.001) and 6 weeks (mean difference 2.234, SD 7.291; *P*=.02) and showed a nonsignificant decrease at 6-months FUP (mean difference 2.466, SD 7.613; *P*=.07). PROMIS depression scores decreased significantly at 1-week (mean difference 4.260, SD 6.811; *P*<.001) and 6-weeks (mean difference 3.175, SD 6.669; *P*<.001) but increased nearly back to baseline at 6 months (mean difference 0.822, SD 6.962; *P*=.50). HIP-10 scores increased significantly at 1 week (mean difference 6.63, SD 12.41; *P*<.001) and 6 weeks (mean difference 6.21, SD 13.37; *P*<.001) and maintained a trend toward an increase at 6 months (mean difference –3.62, SD 12.811; *P*=.12). Table 2 summarizes changes in the scores for Pathways participants relative to their baseline scores.

There were no significant differences in changes in PROMIS anxiety, PROMIS depression, or HIP-10 scores of participants based on time from completion of active treatment to the time of the workshop (≤6 months vs >6 months), stage (stage 0 or 1 vs stage 2 or 3), hormone receptor status (positive vs negative) or nodal status at any follow-up point. Participants with HER2-positive breast cancer displayed a greater decrease in PROMIS depression scores than HER2-negative participants at all FUPs, although the difference was only significant at the 6-week FUP (*P*=.02). On comparing HER2-positive vs -negative participants, there were no significant differences in changes in PROMIS anxiety or HIP-10 scores from baseline to any FUP. Participants who had a shorter treatment duration (≤6 months) displayed a greater decrease in PROMIS anxiety scores than those with a longer treatment duration (>6 months) at all FUPs, although the difference was only significant at the 6-week follow-up (*P*=.049). There were no significant differences in PROMIS depression or HIP-10 scores at any FUP based on treatment length.

The average scores for satisfaction with the workshop and the moderator were 9.70 and 9.96 respectively. 98.5% would recommend the workshop to other survivors. In the immediate feedback provided at the end of the workshop, comments were all favorable and included statements such as: “This program truly gives me a pathway and an orientation of self-care. Instead of being stuck in fear, I have now a way towards a full life” and “The program offers an opportunity for “pause” in a time of great stress caused by dealing with disease and how it upends life…The skills/tools are useful in all aspects of life.”

In responding to the question of what were the most helpful parts of the program, comments included the following:

The constant participation of everyone in the group. It was great to learn from others' experiences. The intentions and appreciations parts were my favorites.

I most enjoyed the recasting, as it provided an intimate listening and sharing setting. I also enjoyed the appreciation line – although it was difficult, it was amazing to see connections had formed in a short space of time.

Some comments regarding areas for improvement were the following:

Would be willing to do two days and/or reconnecting or having a checking in in 3 months/6 months.

A longer program so as to allow the participants more time to share.

Perhaps it could be done in two shorter sessions (3-4 hours each) to give the participants time to reflect on the first session before doing the second-- it's a lot to take in!

**Table 2:** Summary of outcomes among Pathways participants relative to baseline.

Item		Participants, n	Score change			SE		*t* test (*df*)		*P* value
			Mean (SD)	95% CI						
**Patient-Reported Outcomes Measurement Information System anxiety T-score**										
	Baseline to 1-week follow-up	68	3.884 (6.616)	2.282 to 5.485	0.802		4.8409 (67)		<.001	
	Baseline to 6-week follow-up	61	2.234 (7.291)	0.367 to 4.102	0.934		2.3936 (60)		.02	
	Baseline to 6-month follow-up	32	2.466 (7.613)	–0.279 to 5.210	1.346		1.8321 (31)		.08	
**Patient-Reported Outcomes Measurement Information System depression T-score**										
	Baseline to 1-week follow-up	68	4.260 (6.811)	2.612 to 5.909	0.826		5.1583 (67)		<.001	
	Baseline to 6-week follow-up	61	3.175 (6.669)	1.467 to 4.883	0.854		3.7188 (60)		<.001	
	Baseline to 6-month follow-up	32	0.822 (6.962)	–1.688 to 3.332	1.231		0.6678 (31)		.51	
**Happiness Index Profile score**										
	Baseline to 1-week follow-up	68	–6.632 (12.410)	–9.636 to –3.629	1.505		–4.4072 (67)		<.001	
	Baseline to 6-week follow-up	61	–6.213 (13.375)	–9.639 to –2.787	1.713		–3.6280 (60)		<.001	
	Baseline to 6-month follow-up	32	–3.625 (12.811)	–8.244 to 0.994	2.265		–1.6007 (31)		.12	

## Discussion

### Principal Findings

The Pathways for Survivors workshop was well received by patients, and the overwhelming majority would recommend the workshop to other cancer survivors. Participants’ PROMIS anxiety and depression scores decreased significantly up to 6 weeks after the workshop. Improvements in these QOL measures did not appear to differ on the basis of stage, time from the end of active treatment, nodal status, or hormone receptor status. Increased HIP-10 scores at 1-week and 6-week FUPs suggested that patients incorporated the intention and resiliency skills that were the focus of the workshop. While the 6-month FUPs for anxiety and HIP-10 showed a trend toward improvement compared to baseline, these results were not significant, probably owing to the smaller sample size, given that the 6-month FPU survey was only distributed to participants in the last 4 workshops, and a lower percentage (65%) of participants completed the 6-month FUP as compared to the 1- and 6-week FUPs (88 and 80%, respectively). It is also possible that the skills learned in the workshops may need to be reinforced with additional “booster” sessions. A randomized clinical trial of 8 weeks of CBT followed by 3 booster sessions in patients with metastatic breast cancer found sustained reductions in depressive symptoms and anxiety out to 6 months, which supports this hypothesis [21].

Patients who participated in the Pathways workshops, on average, had more anxiety and depression at baseline than a reference population of early-stage patients receiving routine FUP care at the UCSF BCC. Pathways participants were younger, closer to their diagnosis of breast cancer, and had more recently entered the “survivorship” phase of care than the reference group of routine FUP care patients. Notably, many of the Pathways patients were recruited by their medical or surgical oncologist to attend the Pathways workshop, and the providers likely identified patients who they thought had more psychological distress and would benefit from the intervention. Finally, the last 2 workshops were specifically targeted at younger women (<50 years of age), where the additional stresses of having children or returning to the workforce after a cancer diagnosis may be associated with greater anxiety or depression [22,23].

While it is possible that the improvements seen in the Pathways participants over time represents a natural trend of emotional and psychological recovery from the diagnosis of breast cancer and its treatment, the significant decrease in PROMIS anxiety and depression scores and improvement in the HIP-10 scores immediately after the workshop as early as the 1-week time point and sustained until 6 weeks suggests an immediate impact from the workshop. Although the intervention effect size seems to diminish at the 6-month FUP, there are still trends toward decreased anxiety and depression, and improvements in the HIP-10 score—a measure of self-efficacy and tendency toward making positive and intentional behavior choices.

The Pathways for Survivors workshop was based on an “intention model,” which has been applied within numerous business and human resource settings and has been pragmatically refined over time. A pilot study among cardiac rehabilitation patients and their caregivers, also incorporating this “intention model,” revealed more positive attitudes and an improved sense of control and hope related to health, which remained stable at FUP out to 12 weeks [24]. Multiple other positive psychology interventions including mindfulness, expressive writing, and creation of hope have been studied and found to have an overall favorable impact on the QOL of patients with breast cancer [25].

Although a formal mixed methods analysis to evaluate common themes of the feedback was not conducted, participants’ comments reflected that they valued the toolkit of “setting intentions,” exploring obstacles, and incorporating exercises in gratitude and recasting. Participants also rated the group experience as an important aspect of the workshop and reported that the opportunity to interact with other survivors, share experiences, and actively engage in discussions helped bring the concepts to life. Although this was a skills-based workshop, prior research has shown that breast cancer support groups and other forms of peer support provide emotional and informational benefits, although their short- and long-term impact on anxiety and depression is not fully proven [26,27].

Previous studies have supported the efficacy of CBT and mindfulness-based therapies in patients with cancer in addressing FCR, depression, anxiety, and QOL. A meta-analysis of cognitive behavioral interventions among patients with breast cancer undergoing active treatment reported that these techniques had a significant effect in reducing anxiety and depression, and reported that while therapy length or delivery did not significantly moderate the effect, individual therapy showed a slight trend toward eliciting better results on distress outcomes [13]. Another meta-analysis review of mindfulness-based stress reduction programs by Zhang et al [17] reported that most programs were 6-8 weeks long, and had significant effects on anxiety and depression. A randomized controlled trial including breast, prostate, and colorectal cancer survivors receiving 8 sessions of blended CBT revealed a significant decrease in FCR as well as anxiety and depression on the Hospital Anxiety and Depression scale at 3 months from baseline [16]. A pilot study of a 1-2–day psychosocial intervention combining mindfulness-based CBT and covering anxiety management and relationships or sexuality issues for young breast cancer survivors was well received and resulted in an overall gain in self-reported knowledge and confidence among participants [28]. This pilot study led to a much larger randomized controlled trial of a mindfulness-based program compared to survivorship education and a waitlist control for the management of depressive symptoms in younger breast cancer survivors. Our intervention is unique from many previously reported interventions in that it involves only a single session and is led by a lay moderator, making it more convenient and accessible to a population of patients who may find it challenging to attend multiple weekly sessions.

### Limitations

Though the results support an improvement in anxiety, depression, and intention and resiliency as immediately as 1 week after the workshop, suggesting a direct impact of the intervention, as with many previously reported interventional studies that attempted to impact QOL in the survivorship population, this study was not a randomized trial. Nonetheless, we attempted to contextualize the Pathways participants, both in terms of clinical and demographic factors, and baseline anxiety and depression scores in comparison to a reference subpopulation of general early-stage breast cancer FUP patients. However, our routine care population was only sampled at one time point; therefore, we do not have a trajectory of their PROMIS anxiety and depression scores over time and thus does not serve as a true control group. Pathways participants were generally younger than both the average breast cancer survivor as well as our comparison group, and were of a high socioeconomic status (graduate degree holders, annual income of >US $100,000) and working on a full-time basis. As an academic center, we attract a higher risk and younger patient population. While we do see a diverse population, many of our patients are highly educated with high health literacy, who are seeking clinical trials, and are willing to participate in research. These patients are fit enough and have the financial resources to travel for their cancer care. Further research with more heterogeneous patients and with a larger 6-month follow-up sample is needed to confirm that the positive impact on several QOL measures from this positive psychology or mindfulness and skills-based workshop can be sustained and also observed in a more diverse population.

### Clinical Implications

Transitioning from a patient with breast cancer to a breast cancer survivor is associated with a significant burden of psychological distress. Our study supports the Pathways for Survivors workshop as a highly satisfying and time-efficient means for breast cancer survivors to learn behavioral skills and the incorporation of this workshop into survivorship care may help improve emotional well-being and potentially overall QOL.

### Conclusions

The Pathways for Survivors workshop was favorably received, and patients’ anxiety and depression decreased significantly at 1 and 6 weeks after the workshop and remained below baseline at 6 months. While the 1-day workshop format is unique and is more convenient and accessible for patients who may find attending multiple weekly sessions challenging, future research is necessary to explore the impact of integrating of videoconferencing, additional “booster” sessions to reinforce the skills and concepts illustrated in this workshop, and to evaluate its longer-term impact.

## Abbreviations

BCC: Breast Cancer Center
CBT: cognitive behavioral therapy
FCR: fear of cancer recurrence
FUP: follow-up
HIP-10: Happiness Index Profile
PROMIS: Patient-Reported Outcomes Measurement Information System
QOL: quality of life
UCSF: University of California San Francisco
